# Diethyl­enetriaminium hexa­fluorido­titanate(IV) fluoride

**DOI:** 10.1107/S1600536808009781

**Published:** 2008-10-09

**Authors:** J. Lhoste, K. Adil, M. Leblanc, V. Maisonneuve

**Affiliations:** a Laboratoire des Oxydes et Fluorures, UMR 6010 CNRS, Faculté des Sciences et Techniques, Université du Maine, Avenue Olivier Messiaen, 72085 Le Mans Cedex 9, France

## Abstract

The title compound, (C_6_H_21_N_4_)[TiF_6_]F, was synthesized by the reaction of TiO_2_, tris­(2-amino­ethyl)amine, HF and ethanol at 463 K in a microwave oven. The crystal structure consists of two crystallographically independent [TiF_6_]^2−^ anions, two fluoride anions and two triply-protonated tris­(2-amino­ethyl)­amine cations. The Ti atoms are coordinated by six F atoms within slightly distorted octa­hedra. The anions and cations are connected by inter­molecular N—H⋯F hydrogen bonds.

## Related literature

For background, see: Adil *et al.* (2006 ▶). For related structures, see: Calov *et al.* (1992 ▶); Dadachov *et al.* (2000 ▶); Tang *et al.* (2001 ▶).
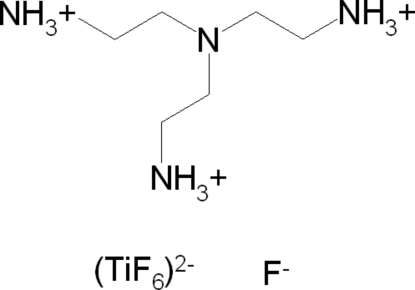


## Experimental

### 

#### Crystal data

(C_6_H_21_N_4_)[TiF_6_]F
*M* *_r_* = 330.14
Monoclinic, 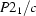

*a* = 16.265 (4) Å
*b* = 8.089 (3) Å
*c* = 21.778 (5) Å
β = 110.54 (2)°
*V* = 2683.1 (13) Å^3^
*Z* = 8
Mo *K*α radiation
μ = 0.71 mm^−1^
*T* = 298 (2) K
0.18 × 0.13 × 0.06 mm

#### Data collection

Siemens AED2 diffractometer
Absorption correction: Gaussian (*SHELX76*; Sheldrick, 2008 ▶) *T*_min_ = 0.850, *T*_max_ = 0.929
6191 measured reflections
6133 independent reflections
3531 reflections with *I* > 2σ(*I*)
3 standard reflections frequency: 120 min intensity decay: 15%

#### Refinement

*R*[*F* ^2^ > 2σ(*F* ^2^)] = 0.061
*wR*(*F* ^2^) = 0.155
*S* = 1.12
6133 reflections
332 parameters
H-atom parameters constrained
Δρ_max_ = 1.37 e Å^−3^
Δρ_min_ = −0.42 e Å^−3^

### 

Data collection: *STADI4* (Stoe & Cie, 1998 ▶); cell refinement: *STADI4*; data reduction: *X-RED* (Stoe & Cie, 1998 ▶); program(s) used to solve structure: *SHELXS97* (Sheldrick, 2008 ▶); program(s) used to refine structure: *SHELXL97* (Sheldrick, 2008 ▶); molecular graphics: *DIAMOND* (Brandenburg, 2001 ▶); software used to prepare material for publication: *enCIFer* (Allen *et al.*, 2004 ▶).

## Supplementary Material

Crystal structure: contains datablocks global, I. DOI: 10.1107/S1600536808009781/nc2095sup1.cif

Structure factors: contains datablocks I. DOI: 10.1107/S1600536808009781/nc2095Isup2.hkl

Additional supplementary materials:  crystallographic information; 3D view; checkCIF report

## Figures and Tables

**Table 1:** Selected bond lengths (Å)

Ti1—F1	1.796 (3)
Ti1—F2	1.826 (3)
Ti1—F4	1.856 (3)
Ti1—F5	1.865 (3)
Ti1—F3	1.868 (3)
Ti1—F6	1.882 (3)
Ti2—F8	1.803 (3)
Ti2—F7	1.821 (3)
Ti2—F9	1.825 (3)
Ti2—F10	1.827 (3)
Ti2—F11	1.832 (3)
Ti2—F12	1.856 (3)

**Table 2:** Hydrogen-bond geometry (Å, °)

*D*—H⋯*A*	*D*—H	H⋯*A*	*D*⋯*A*	*D*—H⋯*A*
N2—H2*C*⋯F13	0.89	1.90	2.773 (5)	165
N2—H2*D*⋯F6^i^	0.89	2.04	2.865 (5)	154
N2—H2*E*⋯F13^i^	0.89	1.84	2.725 (5)	172
N3—H3*C*⋯F13	0.89	1.86	2.700 (5)	157
N3—H3*C*⋯N1	0.89	2.52	2.948 (6)	110
N3—H3*D*⋯F3	0.89	1.90	2.726 (5)	154
N3—H3*E*⋯F9	0.89	1.84	2.717 (5)	167
N4—H4*C*⋯F13	0.89	1.83	2.692 (5)	162
N4—H4*D*⋯F12^i^	0.89	2.01	2.835 (5)	153
N4—H4*E*⋯F5	0.89	1.84	2.712 (5)	168
N6—H6*C*⋯F14	0.89	1.84	2.696 (5)	162
N6—H6*D*⋯F10^ii^	0.89	2.00	2.823 (5)	154
N6—H6*E*⋯F4^iii^	0.89	1.90	2.749 (5)	160
N7—H7*C*⋯F14	0.89	1.82	2.699 (5)	169
N7—H7*D*⋯F2^iii^	0.89	2.24	2.876 (5)	129
N7—H7*E*⋯F7^i^	0.89	2.08	2.916 (5)	157
N7—H7*E*⋯F10^i^	0.89	2.41	2.972 (5)	121
N8—H8*C*⋯F14	0.89	1.91	2.791 (5)	168
N8—H8*D*⋯F6	0.89	2.14	2.879 (5)	140
N8—H8*E*⋯F14^iv^	0.89	1.81	2.702 (5)	177

Symmetry codes: (i) 
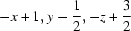
; (ii) 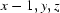; (iii) 
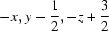
; (iv) 
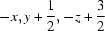
.

**Fig. 1.**
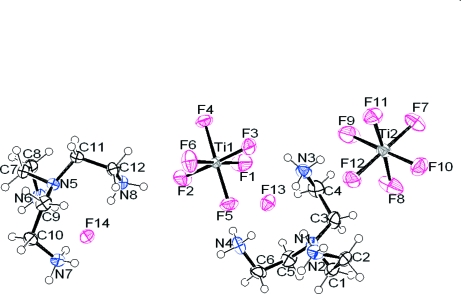
Crystal structure of (I) with displacement ellipsoids for the non-hydrogen atoms drawn at the 50% probability level.

**Fig. 2.**
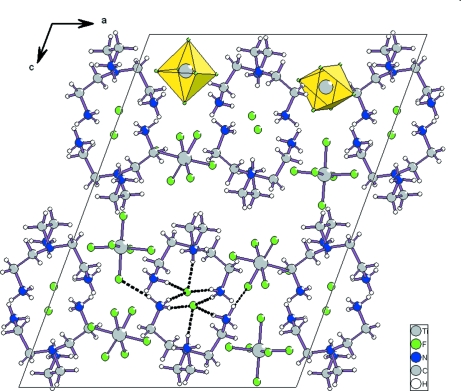
Crystal packing of (I) with view along [010]. Hydrogen bonding is shown as dashed lines.

## supplementary crystallographic information

### Comment

To date, only a few organic-inorganic fluorotitanates were reported. The first compound {(CN_3_H_6_)[TiF_6_]}, described by Calov *et al.* (1992), is built up from (TiF_6_) monomers and guanidinium cations. Dadachov *et al.* (2000) and Tang *et al.* (2001) reported the synthesis of piperazinium {(C_4_N_2_H_12_)_2_[Ti_2_F_10_]2(H_2_O)} and piperidinium {[C_5_H_6_N_2_]2(Ti_2_F_11_)(H_3_O)(H_2_0)} fluorotitanates respectively, built up from (Ti_2_F_10_)^2-^ or (Ti_2_F_11_)^3-^ dimers. As a part of our ongoing investigations in this field (Adil  *et al.*, 2006)) we now report the synthesis and structure of the title compound, (I).

The asymmetric unit of (I) consits of two crystallographically independent (TiF_6_)^2-^ anions, two fluoride anions and two triprotonated tris(2-aminoethyl)amine (tren) cations, all of them located in general positions (Fig. 1).  The TiF_6_ anions form sligthly distorted octahedra and the environnement of both independent anions is different (Table 1). Both TiF_6_ anions are connected to the cations via N—H···F hydrogen bonding (Figure 2 and Table 2).

The two isolated fluoride anions are also hydrogen bonded to the [H_3_tren]^3+^ cations with N—H···F distances ranging from 2.692 (5)Å to 2.791 (5)Å (Figure 2 and Table 2).

### Experimental

The synthetis was performed by using a microwave-assisted route. Crystals were prepared from a mixture of titanium(IV) oxide (79 mg, 1 mmol), tris(2-aminoethyl)amine (0.230 ml, 1.52 mmol), hydrogen fluoride (40%, 0.130 ml, 2.95 mmol) and ethanol (10 ml, 35 mmol). The mixture was transferred into a teflon autoclave installed in a CEM microwave oven at 493 K for 1 hour under a constant pressure of 22 bar. Finally, the solid product was washed with ethanol and dried in air at room temperature to yield colourless paralellepipeds of (I).

### Refinement

A number of mis-measured reflections were omitted from the refinement.

The hydrogen atoms were positioned with idealized geometry (C—H = 0.88-0.97Å, N—H = 0.89Å) and modelled as riding with a group U_iso_ value refined.

The highest difference is 2.49 Å from H4A. It might be that this peak corresponds to a small amount of water, which cannot be proven. Therefore, this electron density was not considered in the final refinement.

### Crystal data

**Table:**

(C_6_H_21_N_4_)[TiF_6_]F	*F*(000) = 1360
*M**_r_* = 330.14	*D*_x_ = 1.635 Mg m^−^^3^
Monoclinic, *P*2_1_/*c*	Mo *K*α radiation, λ = 0.71069 Å
Hall symbol: -P 2ybc	Cell parameters from 32 reflections
*a* = 16.265 (4) Å	θ = 29.1–30.9°
*b* = 8.089 (3) Å	µ = 0.71 mm^−^^1^
*c* = 21.778 (5) Å	*T* = 298 K
β = 110.54 (2)°	Parallelepiped, colourless
*V* = 2683.1 (13) Å^3^	0.18 × 0.13 × 0.06 mm
*Z* = 8	

### Data collection

**Table:**

Siemens AED2 diffractometer	3531 reflections with *I* > 2σ(*I*)
Radiation source: fine-focus sealed tube	*R*_int_ = 0.0000
graphite	θ_max_ = 27.5°, θ_min_ = 2.0°
2θ/ω scans	*h* = −21→19
Absorption correction: gaussian (SHELX76; Sheldrick, 2008)	*k* = 0→10
*T*_min_ = 0.850, *T*_max_ = 0.929	*l* = 0→28
6191 measured reflections	3 standard reflections every 120 min
6133 independent reflections	intensity decay: 15%

### Refinement

**Table:**

Refinement on *F*^2^	Primary atom site location: structure-invariant direct methods
Least-squares matrix: full	Secondary atom site location: difference Fourier map
*R*[*F*^2^ > 2σ(*F*^2^)] = 0.061	Hydrogen site location: inferred from neighbouring sites
*wR*(*F*^2^) = 0.155	H-atom parameters constrained
*S* = 1.12	*w* = 1/[σ^2^(*F*_o_^2^) + (0.0532*P*)^2^ + 2.5558*P*] where *P* = (*F*_o_^2^ + 2*F*_c_^2^)/3
6133 reflections	(Δ/σ)_max_ = 0.001
332 parameters	Δρ_max_ = 1.37 e Å^−^^3^
0 restraints	Δρ_min_ = −0.42 e Å^−^^3^

### Special details

**Table:**

Geometry. All e.s.d.'s (except the e.s.d. in the dihedral angle between two l.s. planes) are estimated using the full covariance matrix. The cell e.s.d.'s are taken into account individually in the estimation of e.s.d.'s in distances, angles and torsion angles; correlations between e.s.d.'s in cell parameters are only used when they are defined by crystal symmetry. An approximate (isotropic) treatment of cell e.s.d.'s is used for estimating e.s.d.'s involving l.s. planes.
Refinement. Refinement of *F*^2^ against ALL reflections. The weighted *R*-factor *wR* and goodness of fit *S* are based on *F*^2^, conventional *R*-factors *R* are based on *F*, with *F* set to zero for negative *F*^2^. The threshold expression of *F*^2^ > σ(*F*^2^) is used only for calculating *R*-factors(gt) *etc*. and is not relevant to the choice of reflections for refinement. *R*-factors based on *F*^2^ are statistically about twice as large as those based on *F*, and *R*-factors based on ALL data will be even larger.

### Fractional atomic coordinates and isotropic or equivalent isotropic displacement parameters (Å^2^)

**Table:**

	*x*	*y*	*z*	*U*_iso_*/*U*_eq_	
Ti1	0.18135 (5)	0.59284 (10)	0.60307 (4)	0.02406 (18)	
Ti2	0.70766 (5)	0.58675 (11)	0.64839 (4)	0.0319 (2)	
F1	0.1489 (3)	0.5816 (4)	0.51539 (14)	0.0687 (10)	
F2	0.07464 (18)	0.5254 (4)	0.60403 (17)	0.0582 (9)	
F3	0.29513 (18)	0.6604 (4)	0.61299 (17)	0.0539 (8)	
F4	0.14665 (19)	0.8126 (3)	0.59720 (15)	0.0469 (7)	
F5	0.2192 (2)	0.3737 (3)	0.61004 (16)	0.0492 (8)	
F6	0.2182 (2)	0.6079 (5)	0.69502 (13)	0.0557 (9)	
F7	0.72742 (19)	0.7113 (4)	0.58528 (14)	0.0518 (8)	
F8	0.6614 (2)	0.4174 (4)	0.59328 (14)	0.0631 (10)	
F9	0.5986 (2)	0.6765 (4)	0.6291 (2)	0.0817 (13)	
F10	0.81774 (18)	0.4997 (4)	0.66930 (15)	0.0494 (8)	
F11	0.7527 (2)	0.7485 (4)	0.70991 (15)	0.0533 (8)	
F12	0.6933 (2)	0.4627 (4)	0.71561 (15)	0.0555 (9)	
N1	0.4459 (2)	0.1509 (5)	0.60450 (17)	0.0276 (8)	
C1	0.5134 (3)	0.0224 (6)	0.6192 (2)	0.0361 (11)	
H1A	0.5186	−0.0159	0.5785	0.044 (2)*	
H1B	0.4952	−0.0708	0.6394	0.044 (2)*	
C2	0.6020 (3)	0.0814 (6)	0.6642 (2)	0.0342 (10)	
H2A	0.6448	−0.0063	0.6703	0.044 (2)*	
H2B	0.6206	0.1748	0.6444	0.044 (2)*	
N2	0.5983 (2)	0.1311 (5)	0.72899 (18)	0.0333 (9)	
H2C	0.5635	0.2188	0.7239	0.044 (2)*	
H2D	0.6521	0.1565	0.7560	0.044 (2)*	
H2E	0.5773	0.0479	0.7458	0.044 (2)*	
C3	0.4511 (3)	0.2578 (6)	0.5509 (2)	0.0392 (12)	
H3A	0.4246	0.2012	0.5093	0.044 (2)*	
H3B	0.5123	0.2784	0.5571	0.044 (2)*	
C4	0.4048 (3)	0.4209 (7)	0.5489 (2)	0.0418 (12)	
H4A	0.4078	0.4863	0.5124	0.044 (2)*	
H4B	0.3434	0.4006	0.5419	0.044 (2)*	
N3	0.4453 (3)	0.5149 (5)	0.61097 (19)	0.0379 (9)	
H3C	0.4595	0.4455	0.6448	0.044 (2)*	
H3D	0.4073	0.5894	0.6150	0.044 (2)*	
H3E	0.4935	0.5660	0.6103	0.044 (2)*	
C5	0.3582 (3)	0.0778 (7)	0.5890 (2)	0.0394 (11)	
H5A	0.3532	−0.0187	0.5615	0.044 (2)*	
H5B	0.3142	0.1572	0.5645	0.044 (2)*	
C6	0.3406 (3)	0.0281 (6)	0.6498 (3)	0.0399 (12)	
H6A	0.2837	−0.0253	0.6373	0.044 (2)*	
H6B	0.3847	−0.0512	0.6743	0.044 (2)*	
N4	0.3420 (2)	0.1714 (5)	0.69203 (18)	0.0375 (10)	
H4C	0.3941	0.2208	0.7036	0.044 (2)*	
H4D	0.3325	0.1373	0.7278	0.044 (2)*	
H4E	0.3002	0.2425	0.6702	0.044 (2)*	
N5	0.0735 (2)	0.5437 (4)	0.89540 (17)	0.0257 (8)	
C7	−0.0032 (3)	0.6180 (6)	0.9046 (2)	0.0303 (10)	
H7A	0.0129	0.7239	0.9263	0.044 (2)*	
H7B	−0.0232	0.5471	0.9324	0.044 (2)*	
C8	−0.0768 (3)	0.6422 (6)	0.8393 (2)	0.0371 (11)	
H8A	−0.1257	0.6977	0.8462	0.044 (2)*	
H8B	−0.0566	0.7112	0.8111	0.044 (2)*	
N6	−0.1063 (2)	0.4800 (5)	0.80724 (18)	0.0396 (10)	
H6C	−0.0602	0.4245	0.8049	0.044 (2)*	
H6D	−0.1447	0.4962	0.7670	0.044 (2)*	
H6E	−0.1316	0.4222	0.8305	0.044 (2)*	
C9	0.1286 (3)	0.4546 (6)	0.9547 (2)	0.0330 (10)	
H9A	0.1348	0.5216	0.9930	0.044 (2)*	
H9B	0.1867	0.4386	0.9526	0.044 (2)*	
C10	0.0905 (3)	0.2884 (6)	0.9620 (2)	0.0324 (10)	
H10A	0.1238	0.2425	1.0047	0.044 (2)*	
H10B	0.0304	0.3030	0.9598	0.044 (2)*	
N7	0.0924 (3)	0.1710 (5)	0.91024 (19)	0.0350 (9)	
H7C	0.0633	0.2141	0.8710	0.044 (2)*	
H7D	0.0673	0.0763	0.9148	0.044 (2)*	
H7E	0.1478	0.1522	0.9138	0.044 (2)*	
C11	0.1253 (3)	0.6685 (5)	0.8757 (2)	0.0309 (10)	
H11A	0.1671	0.7186	0.9147	0.044 (2)*	
H11B	0.0864	0.7548	0.8508	0.044 (2)*	
C12	0.1743 (3)	0.5946 (6)	0.8347 (2)	0.0319 (10)	
H12A	0.2105	0.6789	0.8252	0.044 (2)*	
H12B	0.2126	0.5069	0.8592	0.044 (2)*	
N8	0.1116 (2)	0.5266 (5)	0.77194 (18)	0.0329 (9)	
H8C	0.0850	0.4378	0.7804	0.044 (2)*	
H8D	0.1408	0.4986	0.7458	0.044 (2)*	
H8E	0.0716	0.6030	0.7523	0.044 (2)*	
F13	0.48067 (17)	0.3777 (3)	0.73054 (12)	0.0381 (6)	
F14	0.00692 (17)	0.2652 (3)	0.78508 (13)	0.0362 (6)	

### Atomic displacement parameters (Å^2^)

**Table:**

	*U*^11^	*U*^22^	*U*^33^	*U*^12^	*U*^13^	*U*^23^
Ti1	0.0232 (4)	0.0229 (4)	0.0258 (4)	0.0008 (3)	0.0081 (3)	−0.0022 (3)
Ti2	0.0313 (4)	0.0306 (4)	0.0335 (4)	0.0001 (4)	0.0109 (3)	0.0052 (4)
F1	0.116 (3)	0.052 (2)	0.0285 (15)	0.000 (2)	0.0141 (17)	−0.0044 (16)
F2	0.0296 (15)	0.0481 (19)	0.096 (3)	−0.0058 (14)	0.0206 (16)	0.0027 (18)
F3	0.0365 (16)	0.0480 (18)	0.088 (2)	−0.0072 (14)	0.0353 (17)	−0.0135 (17)
F4	0.0507 (18)	0.0262 (15)	0.064 (2)	0.0073 (13)	0.0211 (15)	−0.0005 (14)
F5	0.0505 (17)	0.0293 (16)	0.066 (2)	0.0112 (13)	0.0185 (15)	0.0061 (14)
F6	0.0519 (18)	0.086 (2)	0.0284 (15)	−0.0037 (18)	0.0125 (13)	−0.0022 (16)
F7	0.0519 (18)	0.0520 (19)	0.0443 (17)	−0.0129 (15)	0.0079 (14)	0.0156 (15)
F8	0.088 (2)	0.055 (2)	0.0351 (16)	−0.0295 (19)	0.0079 (16)	0.0026 (16)
F9	0.0399 (19)	0.046 (2)	0.163 (4)	0.0094 (16)	0.041 (2)	0.025 (2)
F10	0.0447 (17)	0.0482 (19)	0.0568 (18)	0.0151 (15)	0.0196 (15)	0.0040 (16)
F11	0.071 (2)	0.0408 (17)	0.0527 (19)	−0.0096 (16)	0.0270 (17)	−0.0140 (15)
F12	0.094 (2)	0.0410 (17)	0.0477 (18)	−0.0066 (17)	0.0445 (18)	0.0021 (14)
N1	0.0255 (18)	0.0327 (19)	0.0250 (18)	0.0021 (15)	0.0096 (15)	−0.0017 (16)
C1	0.041 (3)	0.031 (2)	0.037 (3)	0.009 (2)	0.015 (2)	−0.002 (2)
C2	0.028 (2)	0.033 (2)	0.043 (3)	0.010 (2)	0.015 (2)	0.007 (2)
N2	0.0265 (19)	0.032 (2)	0.035 (2)	−0.0022 (16)	0.0028 (16)	0.0040 (17)
C3	0.044 (3)	0.052 (3)	0.023 (2)	0.007 (2)	0.014 (2)	0.003 (2)
C4	0.042 (3)	0.050 (3)	0.029 (2)	0.008 (3)	0.008 (2)	0.013 (2)
N3	0.034 (2)	0.034 (2)	0.044 (2)	0.0034 (18)	0.0114 (19)	0.0111 (19)
C5	0.031 (2)	0.044 (3)	0.037 (3)	−0.005 (2)	0.004 (2)	−0.014 (2)
C6	0.033 (2)	0.031 (2)	0.055 (3)	−0.008 (2)	0.015 (2)	0.004 (2)
N4	0.029 (2)	0.052 (3)	0.033 (2)	−0.0063 (19)	0.0137 (17)	0.0057 (19)
N5	0.0284 (18)	0.0223 (18)	0.0283 (18)	−0.0008 (14)	0.0121 (15)	0.0013 (14)
C7	0.032 (2)	0.030 (2)	0.035 (2)	0.0019 (19)	0.0192 (19)	−0.0014 (19)
C8	0.036 (3)	0.037 (3)	0.042 (3)	0.014 (2)	0.018 (2)	0.013 (2)
N6	0.032 (2)	0.053 (3)	0.031 (2)	0.0091 (19)	0.0073 (17)	0.003 (2)
C9	0.030 (2)	0.036 (3)	0.025 (2)	0.0015 (19)	−0.0001 (18)	−0.0033 (19)
C10	0.042 (3)	0.030 (2)	0.024 (2)	0.006 (2)	0.010 (2)	0.0087 (19)
N7	0.045 (2)	0.026 (2)	0.038 (2)	0.0029 (18)	0.0197 (19)	0.0046 (17)
C11	0.036 (2)	0.023 (2)	0.038 (3)	−0.0053 (19)	0.019 (2)	−0.0027 (19)
C12	0.025 (2)	0.031 (2)	0.044 (3)	−0.003 (2)	0.0176 (19)	0.000 (2)
N8	0.039 (2)	0.030 (2)	0.038 (2)	0.0009 (17)	0.0249 (18)	0.0004 (17)
F13	0.0377 (15)	0.0401 (16)	0.0357 (15)	−0.0005 (12)	0.0119 (12)	−0.0088 (12)
F14	0.0398 (15)	0.0338 (15)	0.0352 (14)	−0.0026 (12)	0.0134 (12)	−0.0064 (12)

### Geometric parameters (Å, °)

**Table:**

Ti1—F1	1.796 (3)	C6—N4	1.475 (6)
Ti1—F2	1.826 (3)	C6—H6A	0.9700
Ti1—F4	1.856 (3)	C6—H6B	0.9700
Ti1—F5	1.865 (3)	N4—H4C	0.8900
Ti1—F3	1.868 (3)	N4—H4D	0.8900
Ti1—F6	1.882 (3)	N4—H4E	0.8900
Ti2—F8	1.803 (3)	N5—C7	1.459 (5)
Ti2—F7	1.821 (3)	N5—C11	1.472 (5)
Ti2—F9	1.825 (3)	N5—C9	1.475 (5)
Ti2—F10	1.827 (3)	C7—C8	1.516 (6)
Ti2—F11	1.832 (3)	C7—H7A	0.9700
Ti2—F12	1.856 (3)	C7—H7B	0.9700
N1—C1	1.463 (6)	C8—N6	1.484 (6)
N1—C5	1.470 (6)	C8—H8A	0.9700
N1—C3	1.479 (6)	C8—H8B	0.9700
C1—C2	1.507 (6)	N6—H6C	0.8900
C1—H1A	0.9700	N6—H6D	0.8900
C1—H1B	0.9700	N6—H6E	0.8900
C2—N2	1.487 (6)	C9—C10	1.512 (6)
C2—H2A	0.9700	C9—H9A	0.9700
C2—H2B	0.9700	C9—H9B	0.9700
N2—H2C	0.8900	C10—N7	1.483 (6)
N2—H2D	0.8900	C10—H10A	0.9700
N2—H2E	0.8900	C10—H10B	0.9700
C3—C4	1.512 (7)	N7—H7C	0.8900
C3—H3A	0.9700	N7—H7D	0.8900
C3—H3B	0.9700	N7—H7E	0.8900
C4—N3	1.489 (6)	C11—C12	1.513 (6)
C4—H4A	0.9700	C11—H11A	0.9700
C4—H4B	0.9700	C11—H11B	0.9700
N3—H3C	0.8900	C12—N8	1.494 (6)
N3—H3D	0.8900	C12—H12A	0.9700
N3—H3E	0.8900	C12—H12B	0.9700
C5—C6	1.504 (7)	N8—H8C	0.8900
C5—H5A	0.9700	N8—H8D	0.8900
C5—H5B	0.9700	N8—H8E	0.8900
			
F1—Ti1—F2	94.09 (17)	N1—C5—H5B	109.2
F1—Ti1—F4	90.38 (15)	C6—C5—H5B	109.2
F2—Ti1—F4	91.12 (14)	H5A—C5—H5B	107.9
F1—Ti1—F5	90.21 (15)	N4—C6—C5	111.9 (4)
F2—Ti1—F5	90.17 (14)	N4—C6—H6A	109.2
F4—Ti1—F5	178.55 (14)	C5—C6—H6A	109.2
F1—Ti1—F3	92.71 (17)	N4—C6—H6B	109.2
F2—Ti1—F3	173.16 (16)	C5—C6—H6B	109.2
F4—Ti1—F3	89.59 (14)	H6A—C6—H6B	107.9
F5—Ti1—F3	89.06 (14)	C6—N4—H4C	109.5
F1—Ti1—F6	178.38 (17)	C6—N4—H4D	109.5
F2—Ti1—F6	87.50 (15)	H4C—N4—H4D	109.5
F4—Ti1—F6	89.26 (15)	C6—N4—H4E	109.5
F5—Ti1—F6	90.11 (15)	H4C—N4—H4E	109.5
F3—Ti1—F6	85.71 (15)	H4D—N4—H4E	109.5
F8—Ti2—F7	93.49 (14)	C7—N5—C11	111.2 (3)
F8—Ti2—F9	90.23 (18)	C7—N5—C9	111.6 (3)
F7—Ti2—F9	91.21 (16)	C11—N5—C9	110.8 (3)
F8—Ti2—F10	90.95 (16)	N5—C7—C8	110.9 (4)
F7—Ti2—F10	89.10 (15)	N5—C7—H7A	109.5
F9—Ti2—F10	178.75 (19)	C8—C7—H7A	109.5
F8—Ti2—F11	175.05 (15)	N5—C7—H7B	109.5
F7—Ti2—F11	91.46 (15)	C8—C7—H7B	109.5
F9—Ti2—F11	89.50 (18)	H7A—C7—H7B	108.0
F10—Ti2—F11	89.29 (15)	N6—C8—C7	110.2 (4)
F8—Ti2—F12	88.59 (14)	N6—C8—H8A	109.6
F7—Ti2—F12	177.05 (15)	C7—C8—H8A	109.6
F9—Ti2—F12	90.86 (17)	N6—C8—H8B	109.6
F10—Ti2—F12	88.78 (15)	C7—C8—H8B	109.6
F11—Ti2—F12	86.47 (14)	H8A—C8—H8B	108.1
C1—N1—C5	111.0 (4)	C8—N6—H6C	109.5
C1—N1—C3	110.0 (4)	C8—N6—H6D	109.5
C5—N1—C3	111.9 (4)	H6C—N6—H6D	109.5
N1—C1—C2	112.9 (4)	C8—N6—H6E	109.5
N1—C1—H1A	109.0	H6C—N6—H6E	109.5
C2—C1—H1A	109.0	H6D—N6—H6E	109.5
N1—C1—H1B	109.0	N5—C9—C10	112.4 (3)
C2—C1—H1B	109.0	N5—C9—H9A	109.1
H1A—C1—H1B	107.8	C10—C9—H9A	109.1
N2—C2—C1	110.8 (4)	N5—C9—H9B	109.1
N2—C2—H2A	109.5	C10—C9—H9B	109.1
C1—C2—H2A	109.5	H9A—C9—H9B	107.9
N2—C2—H2B	109.5	N7—C10—C9	111.7 (4)
C1—C2—H2B	109.5	N7—C10—H10A	109.3
H2A—C2—H2B	108.1	C9—C10—H10A	109.3
C2—N2—H2C	109.5	N7—C10—H10B	109.3
C2—N2—H2D	109.5	C9—C10—H10B	109.3
H2C—N2—H2D	109.5	H10A—C10—H10B	107.9
C2—N2—H2E	109.5	C10—N7—H7C	109.5
H2C—N2—H2E	109.5	C10—N7—H7D	109.5
H2D—N2—H2E	109.5	H7C—N7—H7D	109.5
N1—C3—C4	111.6 (4)	C10—N7—H7E	109.5
N1—C3—H3A	109.3	H7C—N7—H7E	109.5
C4—C3—H3A	109.3	H7D—N7—H7E	109.5
N1—C3—H3B	109.3	N5—C11—C12	112.0 (4)
C4—C3—H3B	109.3	N5—C11—H11A	109.2
H3A—C3—H3B	108.0	C12—C11—H11A	109.2
N3—C4—C3	111.2 (4)	N5—C11—H11B	109.2
N3—C4—H4A	109.4	C12—C11—H11B	109.2
C3—C4—H4A	109.4	H11A—C11—H11B	107.9
N3—C4—H4B	109.4	N8—C12—C11	110.7 (3)
C3—C4—H4B	109.4	N8—C12—H12A	109.5
H4A—C4—H4B	108.0	C11—C12—H12A	109.5
C4—N3—H3C	109.5	N8—C12—H12B	109.5
C4—N3—H3D	109.5	C11—C12—H12B	109.5
H3C—N3—H3D	109.5	H12A—C12—H12B	108.1
C4—N3—H3E	109.5	C12—N8—H8C	109.5
H3C—N3—H3E	109.5	C12—N8—H8D	109.5
H3D—N3—H3E	109.5	H8C—N8—H8D	109.5
N1—C5—C6	112.0 (4)	C12—N8—H8E	109.5
N1—C5—H5A	109.2	H8C—N8—H8E	109.5
C6—C5—H5A	109.2	H8D—N8—H8E	109.5

### Hydrogen-bond geometry (Å, °)

**Table:**

*D*—H···*A*	*D*—H	H···*A*	*D*···*A*	*D*—H···*A*
N2—H2C···F13	0.89	1.90	2.773 (5)	165
N2—H2D···F6^i^	0.89	2.04	2.865 (5)	154
N2—H2E···F13^i^	0.89	1.84	2.725 (5)	172
N3—H3C···F13	0.89	1.86	2.700 (5)	157
N3—H3C···N1	0.89	2.52	2.948 (6)	110
N3—H3D···F3	0.89	1.90	2.726 (5)	154
N3—H3E···F9	0.89	1.84	2.717 (5)	167
N4—H4C···F13	0.89	1.83	2.692 (5)	162
N4—H4D···F12^i^	0.89	2.01	2.835 (5)	153
N4—H4E···F5	0.89	1.84	2.712 (5)	168
N6—H6C···F14	0.89	1.84	2.696 (5)	162
N6—H6D···F10^ii^	0.89	2.00	2.823 (5)	154
N6—H6E···F4^iii^	0.89	1.90	2.749 (5)	160
N7—H7C···F14	0.89	1.82	2.699 (5)	169
N7—H7D···F2^iii^	0.89	2.24	2.876 (5)	129
N7—H7E···F7^i^	0.89	2.08	2.916 (5)	157
N7—H7E···F10^i^	0.89	2.41	2.972 (5)	121
N8—H8C···F14	0.89	1.91	2.791 (5)	168
N8—H8D···F6	0.89	2.14	2.879 (5)	140
N8—H8E···F14^iv^	0.89	1.81	2.702 (5)	177

Symmetry codes: (i) −*x*+1, *y*−1/2, −*z*+3/2; (ii) *x*−1, *y*, *z*; (iii) −*x*, *y*−1/2, −*z*+3/2; (iv) −*x*, *y*+1/2, −*z*+3/2.
